# Rationale for Targeting Deregulated Metabolic Pathways as a Therapeutic Strategy in Acute Myeloid Leukemia

**DOI:** 10.3389/fonc.2019.00405

**Published:** 2019-05-22

**Authors:** Nicolas Chapuis, Laury Poulain, Rudy Birsen, Jerome Tamburini, Didier Bouscary

**Affiliations:** ^1^INSERM U1016, Institut Cochin, Paris, France; ^2^CNRS UMR8104, Paris, France; ^3^Université Paris Descartes, Faculté de Médecine Sorbonne Paris Cité, Paris, France; ^4^Equipe Labellisée Ligue Nationale Contre le Cancer (LNCC), Paris, France; ^5^Assistance Publique-Hôpitaux de Paris, Hôpitaux Universitaires Paris Centre, Service d'Hématologie Biologique, Paris, France; ^6^Assistance Publique-Hôpitaux de Paris, Hôpitaux Universitaires Paris Centre, Service d'Hématologie Clinique, Paris, France

**Keywords:** hematopoiesis, acute myeloid leukemia, metabolism, targeted therapy, glycolysis, fatty acid oxidation, oxidative phosphorylation, amino-acid

## Abstract

Metabolic reprogramming is a common cancer cell phenotype as it sustains growth and proliferation. Targeting metabolic activities offers a wide range of therapeutic possibilities which are applicable to acute myeloid leukemia (AML). Indeed, in addition to the IDH1/2-mutated AML model which established the proof-of-concept for specifically targeting metabolic adaptations in AML, several recent reports have expanded the scope of such strategies in these diseases. This review highlights recent findings on metabolic deregulation in AML and summarizes their implications in leukemogenesis.

Acute myeloid leukemias (AMLs) are characterized by abnormal proliferation/survival of hematopoietic progenitors blocked in their differentiation. Recent advances in AML biology, especially in the field of their mutational landscape led to the development of targeted therapies (1). It was unexpected however that the targeting of metabolic pathways could become a potent therapeutic strategy for AML, since such deregulations are highly common in various cancer cells (2–4). However, an improved understanding of the implication of metabolism deregulation in leukemogenesis has led to this renewed interest. The identification of IDH1/2 mutations in AML cells represented the proof-of-concept that deciphering metabolic abnormalities could lead to therapeutic applications in AML. These mutations lead to the abnormal conversion of α-ketoglutarate (α-KG) to (R)-2-hydroxyglutarate [(R)-2HG] which acts as an oncometabolite by inhibiting functions of α-KG-dependent enzymes such as TET family (5). These mutations induce therefore alterations in the pattern of histone modifications and aberrant DNA methylation (6) but have also significant impact on cellular metabolism such as inhibition of the activity of cytochrome c oxidase (COX) in the mitochondrial electron transport chain (ETC) (7) or increased glutamine dependency of AML cells for survival (8). Targeting IDH1/2 mutations in AML is a promising metabolic targeted therapy since inhibitors of mutant-IDH1/2 enzymes induce hematological responses in patients with relapsed/refractory AML by promoting the differentiation of leukemic cells (9, 10). As finding a good therapeutic window between cancer cells and normal cells for the safe administration of new compounds remains a major challenge in the development of therapies, targeting metabolic addictions which are specific of leukemic cells or mechanisms underlying these processes clearly represent a feasible option that may work as an AML therapy. However, metabolism also regulates key processes in normal cells, including hematopoiesis.

This review will therefore highlight the implication of metabolism in normal hematopoiesis and then will focus on the pathways recently reported to be deregulated and potentially targetable in AML.

## Metabolic Regulation of Normal Hematopoiesis

Metabolism supports a set of flexible processes that allow energy production in cells according to their needs which are highly dependent on their state of proliferation, differentiation or quiescence. Hematopoietic stem cells (HSCs) are totipotent cells which ensure the maintenance of the blood system. The balance between HSC self-renewal and lineage differentiation is mainly regulated by transcription factors and cytokines but cell metabolism is also a key player of these processes. In order to maintain their survival and their quiescent state in the hypoxic niche, HSCs adapt their metabolism (11). This low oxygen microenvironment stabilizes hypoxia inducible factors (HIF-1s) and promotes utilization of glycolysis rather than oxidative phosphorylation (OXPHOS) through transcriptional activation of genes that regulate glucose uptake (Glut1) and pyruvate disposal (LDHA and Pdk1). This glycolytic phenotype of HSCs appears to be dependent upon transcriptional activation of HIF-1α through the HSC transcription factor MEIS1 (12). Although ATP generation derived from glycolysis is limited compared to OXPHOS, this metabolism is sufficient to meet the low energy demand of HSCs and is also crucial for their maintenance in a quiescent and functional state. Indeed, given that mitochondrial OXPHOS is a high source of free radical production, relying mostly on glycolysis also prevents HSCs from cellular damage mediated by mitochondria derived ROS. This preferential utilization of glycolysis toward OXPHOS is also mediated by HIF-1. Indeed, HIF-1 stimulates the expression of pyruvate dehydrogenase kinase (PDK) 2 and 4 which actively prevent pyruvate from entering the TCA cycle and engaging mitochondrial OXPHOS through inhibition of pyruvate dehydrogenase (PDH) (13). It has also been reported a crucial role of PTPMT1, a PTEN-like mitochondrial phosphatase, in the metabolic regulation of HSCs function as its inhibition enhances glycolysis, attenuates OXPHOS and significantly increases HSCs number (14). In this way, blocking glycolysis by deleting enzymes such as PKM2 and LDHA induced a consequent elevated mitochondrial respiration and efficiently abolished HSCs maintenance (15). Others recent findings which revealed that steroid receptor coactivator-3 (SRC-3) is essential for maintenance of HSCs homeostasis via repression of mitochondrial biogenesis also corroborated the need for HSCs to suppress mitochondrial OXPHOS for the maintenance of their quiescence and self-renewal capacities (16). However, although decreased mitochondrial activity in HSCs is clearly associated to preservation of HSCs functions (17–19), functional mitochondria are also required for HSCs maintenance. Indeed, HSCs survival in a mouse mutant with inducible deletion of the mitochondrial protein-encoding SdhD gene which encodes one of the subunits of the mitochondrial complex II (MCII) is impaired (20). Similarly, fumarate hydratase (Fh1), a key component of the mitochondrial tricarboxylic acid (TCAA) cycle and cytosolic fumarate metabolism is also essential for HSCs maintenance (21). Interestingly, fatty acid oxidation (FAO) seems to be a key metabolic process which determines cell fates of HSCs and regulates the balance between maintenance and commitment in dividing cells. FAO is regulated in HSCs by the promyelocytic leukemia (PML) tumor suppressor, protein which activates a family of nuclear receptors called peroxisome proliferator-activated receptors (PPAR). Inhibition of FAO induces HSCs loss and accumulation of committed progenitors (22). This FAO inhibition-mediated differentiation of HSCs is linked to a decrease of asymmetric division in favor of symmetric divisions producing two committed progenitors instead one committed daughter cell and one daughter cell with self-renewal potential, leading therefore to HSC exhaustion. These metabolic processes implicated in cell fate decisions of HSCs are also under the control of bioenergetics sensors which activate signaling pathways according to nutrient availability. For instance, LKB1 and its downstream target AMPK maintains HSCs potential by inducing the transcription of genes encoding FAO enzymes (23).

**Figure 1:**
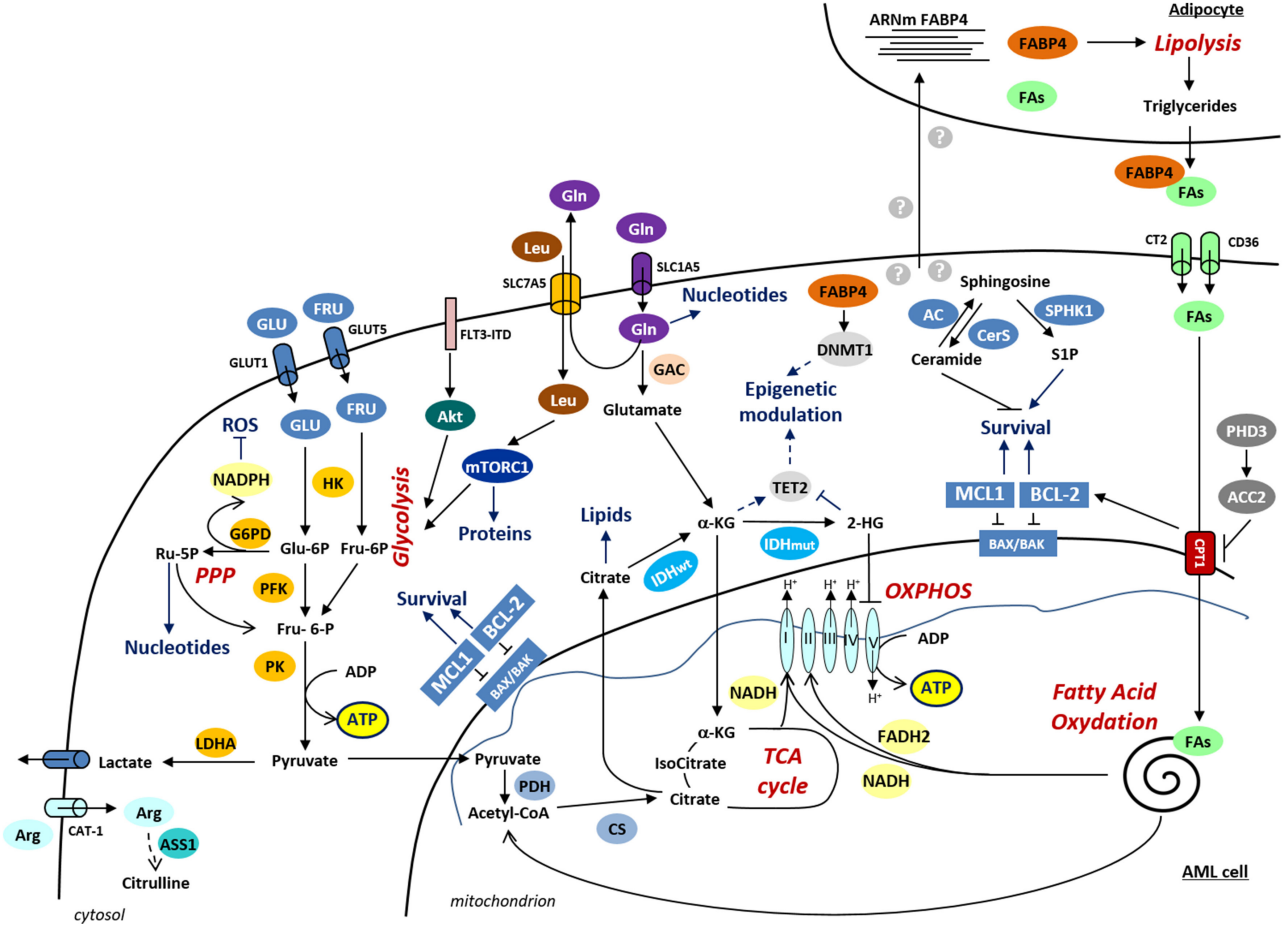
Schematic representation of the deregulated metabolic pathways in AML. Metabolic pathways are indicated in red and the cellular processes regulated by metabolic pathways are denoted in blue. α-KG, α-ketoglutarate; ASS1, argininosuccinate synthetase-1; Arg, arginine; CAT-1, cationic amino acid transporter-1; CS, citrate synthase; CT2, carnitine transporter 2; CTP1, carnitine palmitoyl transferase 1A; DNMT1, DNA methyltransferase 1; FABP4, fatty acid binding protein-4; FAs, fatty acids; FLT3-ITD, FLT3 receptor with internal tandem duplication mutation; FRU, fructose; GAC, glutaminase C; G6PD, glucose-6-phosphate dehydrogenase; Gln, glutamine; GLU, glucose; GLUT1, glucose transporter 1; GLUT5, glucose transporter 5; HK, hexokinase; IDH, isocitrate dehydrogenase; LDHA, lactate dehydrogenase A; Leu, leucine; PDH, pyruvate dehydrogenase; PFK, phosphofructokinase; PK, pyruvate kinase; PPP, pentose phosphate pathway; Ru-5P, Ribulose-5-Phosphate; TET, ten–eleven translocation 2; 2- HG, 2-hydroxyglutarate.

Consistently with the important role of the balance between different metabolic pathways in the regulation of HSC maintenance or differentiation, metabolic dysfunctions are also involved in leukemogenesis (Figure 1). Metabolomics and transcriptomics analysis identified significant differences in the serum metabolic phenotypes and in the metabolic gene expression profile between AML and normal cells, implicating glycolysis/gluconeogenesis, TCA cycle, biosynthesis of proteins and lipoproteins or metabolism of fatty acids (24–31).

## Deregulation of the Glycolytic Pathway in AML

Glycolysis is known to be increased in a high range of tumors cells since Otto Warburg observed the capacity of these cells to favoring production of lactate rather than allowing pyruvate entry into the TCA cycle for producing high rate of ATP (3, 4). Accordingly, glycolysis is increased in most cases of AML (27, 32). High glycolytic activity is implicated in the leukemogenesis driven by BCR-ABL and MLL-AF9 oncogenes since leukemia initiation is inhibited by deleting genes encoding either PKM2 or LDHA (15). Interestingly, leukemia-initiating cells are dependent on a high glycolytic flux mediated by AMPK activation and AMPK deletion suppresses leukemic cells by compromising the glucose flux through a reduced expression of the GLUT1 transporter, and by increasing oxidative stress and DNA damage (33). A high glycolysis flux also correlates with a decreased level of autophagy leading to a more aggressive leukemias *in vivo* (34). Increased glycolysis is also associated with drug resistance (27, 35), notably through the cytosolic export of PCNA (proliferating cell nuclear antigen) which interacts with nicotinamide phosphoribosyltransferase (NAMPT), a protein involved in NAD biosynthesis and coordinates glycolysis and survival in chemo-resistant cells (36). Targeting glycolysis may be therefore a viable strategy for modulating chemo-resistance.

High mTORC1 activity and FLT3-ITD-mediated signaling sustain glycolysis and render AML cells dependent on this pathway for survival and sensitive to its inhibition (37, 38). We described that AML survival relies on glycolysis mainly for sustaining a high flux of glucose through the pentose phosphate pathway, making glucose-6-phosphate dehydrogenase (G6PD) a promising therapeutic target (37). G6PD overexpression correlates with an adverse prognosis in AML and anti-leukemic activity has been observed with the G6PD inhibitor 6-AN (37, 39). The inactivation of G6PD was also identified as a potent therapeutic strategy for FLT3-ITD-mutated AML, due to a synthetic lethality with FLT3 inhibitors (40).

Moreover, the capacity of AML cells to adapt their metabolism to an environment that they modify itself through their high rate of glucose consumption is important. Glucose insufficiency mediated by AML proliferation in the bone marrow micro-environment (BMME) forces AML cells to up-regulate GLUT5 transporter expression, allowing them to use fructose as alternative substrate for glycolysis. A blockage of fructose uptake reduces leukemogenesis and potentiates the cytotoxicity of cytarabine (41). Finally, leukemic cells also manage their increased need for glucose independently of intrinsic mechanisms. By inducing a “diabetic state” in the host through regulation of different mechanisms converging to the suppression of insulin secretion, they allow an increase availability of glucose to drive their own growth. The restoration of normal glucose regulation is a suggested strategy to suppress the systemic growth of leukemic cells (42).

Glycolysis is thus a frequently upregulated metabolic pathway to which AML cells become dependent for their growth, creating therefore a metabolic vulnerability which can be therapeutically exploited.

## Deregulation of Oxidative Phosphorylation and Fatty Acid Oxidation Metabolism in AML Cells

Although oxidative phosphorylation (OXPHOS) is the most efficient mechanism for energy generation, its relative slowness and propensity to induce a high level of ROS production during mitochondrial ATP production leads cancer cells to mainly rely on aerobic glycolysis. Notably however, cancer cell subsets with different dependencies in terms of energy-generating pathways can coexist within tumors. Accordingly, leukemic stem cells (LSCs), in contrast to normal hematopoietic stem cells (HSCs), are deficient in glycolysis use and depend upon BCL2-mediated oxidative respiration for maintenance, suggesting that a selective eradication of these cells may be possible by targeting OXPHOS with BCL2 inhibitors (43). Direct targeting of OXPHOS with metformin also induces AMPK-independent apoptosis by decreasing electron transport chain complex I activity (44). IACS-010759, a small-molecule inhibitor of complex I of the mitochondrial ETC was shown to inhibit AML cells growth *in vivo* and is currently evaluated in relapsed/refractory AML (NCT02882321) (45). Furthermore, AML cells have a lower spare reserve capacity in the respiratory chain and are more susceptible to oxidative stress compared with normal hematopoietic cells. This vulnerability could be therefore therapeutically exploited by compounds such as the fatty acid palmitate which induce oxidative stress and selective AML cell death (46).

Plasticity of cancer cells allowing metabolic adaptations to balance between glycolysis and oxidative metabolism is frequently observed. In AML, mTORC1 inhibition induces metabolic reprograming from a glycolytic to an oxidative process by promoting the TCA cycle through an increase of acetyl-CoA (instead of lactate) production from pyruvate. This leads to a switch of AML cells sensitivity from glycolysis to OXPHOS inhibition, which was therapeutically exploited by concomitant mTORC1 and OXPHOS inhibition (37). Metabolic adaptations likely differ between AML sub-types and also within leukemic cells from the same patient, and could be at the origin of chemo-resistance. Indeed, under cytarabine treatment, drug-resistant AML cells are characterized by a high OXPHOS status and targeting their mitochondrial functions induces an energetic shift toward low OXPHOS and markedly enhances the anti-leukemic effects of cytarabine (47). High OXPHOS activity in AML cells exposed to chemotherapy is supported by an increase of FAO (47) but also depends on PDH activity (48).

The FAO pathway contributes to the plasticity of cancer cell metabolism by allowing ATP production through OXPHOS, the *de novo* synthesis of lipid membrane components and the elimination of potentially toxic lipids (49). In AML, carnitine palmitoyl transferase 1A (CPT1A), which catalyzes the rate-limiting step of FAO, and carnitine transporter CT2 (SLC22A16) are overexpressed and constitute novels targets for a subset of AML (50, 51). Furthermore, AML cells harbor low levels of prolyl-hydroxylase 3 (PHD3) which represses FAO by activating ACC2 during nutrient abundance. Hence, low PHD3 levels in AML cells maintain a high FAO levels regardless of external nutrient availability to drive AML cell proliferation (52). Blocking FAO with ST1326, a specific CPT1A inhibitor, or with avocatin B reduces cell growth and induces AML cells apoptosis without affecting normal CD34+ hematopoietic cells (53, 54). Furthermore, as FAO promotes AML survival by negatively regulating the activity of the Bak-dependent mitochondrial permeability transition, combination with BCL2 inhibitors may improve the susceptibility of AML cells to FAO inhibition (55).

Another possible mechanism for targeting lipid metabolism in AML is to restore the level of Dendrogenin A (DDA), a dropped mammalian cholesterol metabolite. DDA is a partial agonist on liver-X-receptor (LXR) which induces lethal autophagy *in vitro* and *in vivo* in AML independently of their molecular and cytogenetic characteristics (56). Sphingolipids play a critical role in AML in the regulation of the signal balance between cell proliferation/survival and death. Sphingosine 1-phosphate (S1P) and ceramide, two different central bioactive lipids, have opposite roles in regulating cancer cell death and survival. The overexpression of acid ceramidase (AC) which catalyzes the breakdown of ceramide to sphingosine, the precursor for S1P, contributes to AML cell proliferation via the regulation of sphingolipid levels and MCL-1. Targeting AC with the LCL204 compound induces AML cell apoptosis and reduces leukemogenesis *in vivo* (57). AML cells are also sensitive to sphingosine kinase 1 (SPHK1) inhibition, a constitutively activated enzyme which generates S1P (58–60). Interestingly, as a decrease in MCL1 levels is implicated in the SPHK1 inhibition mediated-apoptosis of AML cells, combination of sphingolipids targeted therapy with BH3 mimetics could be beneficial (58). Finally, ceramide generation is inhibited in AML cells harboring FLT3-ITD. The restoration of ceramide generation via ceramide synthase 1 (CerS1) is a key mechanism implicated in AML cell death induced by FLT3 TKI, and also provides an opportunity to overcome resistance to such targeted therapy (61).

Altogether, OXPHOS and mitochondrial ATP production are key metabolic processes to which different frequently deregulated metabolic pathways such as FAO converge. Directly targeting OXPHOS or through indirect means by blocking upstream metabolic pathways constitute therefore an attractive therapeutic strategy whose efficiency could be increased by the concomitant use of BH3 mimetics.

## Targeting the Microenvironment Supporting AML Metabolism

Special attention has recently been paid to the metabolic interactions of AML cells with the BM ME. In a murine model of blast crisis of chronic myeloid leukemia (CML), Ye et al. identified a subpopulation of LSCs that expressed the fatty acid transporter CD36, which is enriched in the adipocyte tissue, allowing these cells to become chemo-resistant. Mechanistically, LSCs induce lipolysis in adipocytes which, in turn, creates a ME that supports LSCs maintenance by fueling their FAO (62). Notably, AML cells enhance lipolysis of adipocytes by upregulating the levels of fatty acid binding protein-4 (FABP4). Hence, fatty acids released by adipocytes drive FAO in AML cells in a CPT1a-dependent manner which facilitates their survival (63, 64). The FABP4 mRNA level is also increased in AML blasts and a knockdown of FABP4 in a Hoxa9/Meis1-driven murine leukemia model prolongs survival (63). Furthermore, FABP4 up-regulation induces IL-6/STAT3-mediated DNMT1 overexpression and the silencing of the p15INK4B tumor-suppressor gene in AML cells, thus confirming that FABP4 disruption may be a viable therapeutic strategy (65). For unknown reasons, this interplay with adipocytes is specific to AML cells. The BMME also supports AML cell metabolism through the transfer of mitochondria, which rescue respiration and cell survival after exposure to chemotherapy (66). AML cells, through the NADPH oxidase-2 (NOX2)-dependent generation of ROS, stimulate the formation by BM stromal cells of tunneling nanotubes with the membrane of AML cells. The inhibition of NOX2, by preventing mitochondrial transfer, increases AML apoptosis and improves AML survival *in vivo* whilst sparing non-malignant CD34+ cell survival (67).

## Targeting the Amino-Acid Addiction of AML Cells

With glucose, glutamine is the main nutrient source for the growth/survival of cancer cells. Glutamine supports tumor growth through its transformation to α-KG, which feeds the TCA cycle, and by contributing to leucine import into the cells, which activates the amino-acid/Rag/Rac/mTORC1 signaling pathway (68). L-asparaginase (L-ase), in addition to its ability to reduce asparagine plasma levels via deamination, also has glutaminase activity which is required for its anti-leukemic activity in AML (69). Glutamine removal or knockdown of its high-affinity transporter SLC1A5 inhibits mTORC1 signaling and induces apoptosis (69). Glutamine also controls mitochondrial OXPHOS and blocking its conversion to glutamate by specific inhibition of Glutaminase C with the CB-839 compound shows anti-leukemic activity (70–72). This strategy is currently being tested in AML in a clinical trial (NCT02071927). Furthermore, as glutaminolysis inhibition activates mitochondrial apoptosis, a synergetic effect is observed with BCL2 inhibitors (71). A previous CRISPR/Cas9 lethality screen of MOLM-13 AML cells treated with an FLT3 TKI also revealed that increased glutaminolysis mediates TKI resistance (73). This relevant metabolic dependency of FLT3-ITD+ AML cells on glutaminolysis is targetable with cooperation of FLT3-ITD and glutaminase inhibitors to prolong survival in mice with AML (74).

AML cells are also dependent on exogenous arginine due to their deficiency in the argininosuccinate synthetase-1 (ASS1) enzyme, which allows cells to synthesize arginine from citrulline. This deficiency confers a proliferative advantage to cancer cells but also a selective addiction of these cells to the extracellular availability of this amino acid which can be exploited by arginine-depleting agents such as pegylated arginine deiminase (ADI-PEG20) (75). Clinical trials are ongoing (NCT01910012 and NCT02875093) in this regard but have thus far demonstrated the capacity of ADI-PEG20 to induce complete remissions or the control of the disease in only some patients suggesting that ASS deficiency is not a sufficient condition for response to ADI-PEG20 monotherapy in AML (76).

## Eradication of LSCs by Targeting Their Metabolic Dependencies

A crucial field of research is to find how to eradicate LSCs by targeting metabolic deregulations. As mentioned earlier, LSCs harbor a selective dependence on OXPHOS which makes them sensitive to BCL2 inhibition (43) whereas HSCs mainly rely on glycolysis (12–15). This greater reliance of LSCs on OXPHOS can be exploited by targeting ClpP, a mitochondrial protease overexpressed in a subset of AML and stem cells that interacts with mitochondrial respiratory chain proteins and metabolic enzymes (77). A proteomic analysis of LSCs has also revealed a high level of branched-chain amino acid transaminase 1 (BCAT1), a critical negative regulator of the intracellular α-KG level, which leads to a DNA hypermethylated state through altered TET activity, therefore mimicking the effects of IDH1/2 mutations (78). Finally, metabolomic studies have revealed a mandatory increase in amino acid metabolism in LSCs. In contrast to non-LSCs and normal HSCs, LSCs are unable to use another metabolic process such as FAO to maintain a sufficient level of OXPHOS for survival in a context of amino acid starvation (79). This lack of metabolic flexibility is therefore an attractive target for developing specific therapies against LSCs. This concept was confirmed in older patients with AML, in which the combination of venetoclax with azacitidine eradicated LSCs by decreasing amino acid uptake, which explains the clinical benefit observed with this strategy (80).

## Conclusion

Metabolic pathways are deregulated in AML cells and play a critical role in leukemogenesis, contributing to chemoresistance and disease relapse. These mechanisms constitute therefore potent targets for AML treatment. However, metabolic abnormalities are multiple and heterogeneous among AML patients. Furthermore, the high plasticity of AML cells that enables them to adapt their metabolism and ensure their survival under selective inhibition of some pathways, and interactions of these cells with the BMME supporting their metabolic pathways, must be taken into account in the development of therapeutic strategies. Targeting specific metabolic addictions of leukemic cells must be privileged. In this regard, a very promising strategy is the synergistic effect observed with BCL2 inhibitors and hypomethylating agents (80, 81). Given that the anti-leukemic activity of these compounds has already been demonstrated, a combination of these agents with the inhibition of specific metabolic pathways converging at the mitochondria is likely to be actually the most potent strategy for AML.

## Author Contributions

All authors listed have made a substantial, direct and intellectual contribution to the work, and approved it for publication.

### Conflict of Interest Statement

The authors declare that the research was conducted in the absence of any commercial or financial relationships that could be construed as a potential conflict of interest.
